# First evidence of bighead carp wild recruitment in Western Europe, and its relation to hydrology and temperature

**DOI:** 10.1371/journal.pone.0189517

**Published:** 2017-12-12

**Authors:** Marco Milardi, Duane Chapman, Mattia Lanzoni, James M. Long, Giuseppe Castaldelli

**Affiliations:** 1 Independent Researcher, Asiago, Italy; 2 U.S. Geological Survey, Columbia Environmental Research Center, Columbia, MO, United States of America; 3 Department of Life Sciences and Biotechnology, University of Ferrara, Ferrara, Italy; 4 U.S. Geological Survey, Oklahoma Cooperative Fish and Wildlife Research Unit, Oklahoma State University, Stillwater, OK, United States of America; Central Michigan University, UNITED STATES

## Abstract

Bighead carp (*Hypophthalmichthys nobilis*) have been introduced throughout Europe, mostly unintentionally, and little attention has been given to their potential for natural reproduction. We investigated the presence of young-of-the-year bighead carp in an irrigation canal network of Northern Italy and the environmental conditions associated with spawning in 2011–2015. The adult bighead carp population of the canal network was composed by large, likely mature, individuals with an average density of 45.2 kg/ha (over 10 fold more than in the main river). The 29 juvenile bighead carp found were 7.4–13.1 cm long (TL) and weighed 9.5–12.7 g. Using otolith-derived spawning dates we estimated that these juveniles were 94–100 days old, placing their fertilization and hatch dates in mid-to-end-June. Using this information in combination with thermal and hydraulic data, we examined the validity of existing models predicting the onset of spawning conditions and the viability of egg pathways to elucidate spawning location of the species. While evidence of reproduction was not found every year, we determined that potentially viable spawning conditions (annual degree-days and temperature thresholds) and pathways of egg drift suitable for hatching are present in short, slow-flowing canals.

## Introduction

The bighead carp (*Hypophthalmichthys nobilis*) is a cyprinid fish native to South and Central China where it thrives in large rivers and associated floodplains. Bighead carps are primarily pelagic filter feeders, preferentially consuming zooplankton, but also phytoplankton and detritus [1–4]. Due to its rapid growth rates, it was the 7th most intensively cultured fish species worldwide in 2014 [5]. Because of its importance in aquaculture in China, bighead carp, along with silver carp (*H*. *molitrix)* grass carp (*Ctenopharyngodon idella*) and black carp (*Mylopharyngodon piceus*) are considered the “four famous domestic fishes”. Herein, we follow many other authors in referring to this group of fishes as the “Asian carps”, though there exist many species of carp in Asia. As with the other three Asian carps, bighead carp have been widely introduced outside their native range via unintentional escapes, biomanipulation programs, or for fishery enhancement [6]. The species is considered undesirable and a detrimental invasive species in many parts of its introduced range [7–10].

Probably the most notable of these invasions is occurring in North America. Bighead carp were initially imported to Arkansas in the early 1970s and were used in aquaculture, and sometimes sewage treatment, in many other states. Bighead carp have since escaped and become established throughout the central and southern portions of the Mississippi River Basin, as well as having been reported in 24 states and in Lake Erie [11]. Bighead carp can outcompete native larval fishes and mussels for plankton resources [12], and potentially affect the entire ecosystem through a series of trophic cascades [13–15].

Bighead carp effects on the ecosystem are still largely unknown in Europe, where the species has also been introduced. Some sources claim that the species is established at least in the Danube River, e.g. [6,16,17], but others report they are only present in Europe through stocking and escapes [18]. In Western Europe, bighead carp was introduced at the beginning of the 1960s and is commonly considered unable to reproduce due to the limited length of its rivers, but there have been no studies on their population stability or their effects on the ecosystem. However, in many instances, bighead carp invasions likely are underreported in favor of silver carp (*Hypophthalmichthys molitrix)*, which are often co-occurring and have a tendency to jump out of the water when boats pass [9], increasing the reporting frequency. These reasons all likely contribute to the lack of studies and management plans for bighead carp in Western Europe, over the more than 50 years since the first introduction.

Areas with high anthropogenic modification, where propagule pressure is strongest or where alien species introductions occurred first, are most likely to show signs of establishment [19–21]. One of the earliest introductions in West Europe was in Italy, where bighead carp were reported as early as 1975 [22]. Because there are no records of authorized stocking in open waters, the species probably was introduced through escapes from aquaculture or unintentional introductions when stocking other Asian carps, especially grass carp [23]. Unintentional introductions such as these are currently not investigated and are a likely vector for the spread of bighead carp in the Po River system, the largest river in Italy and potentially suitable for establishment of the species.

Many studies have focused on reproductive biology and early life history of Asian carps, either for sustainable management as foodfishes [24,25] or to identify the minimum suitable conditions for establishment and model their spread where they are considered undesirable [26–28]. The reproduction and early life history of Asian carps is thus well described, and is similar among the species. These rheophilic spawners typically migrate upstream to locations of high water velocity and turbulence for spawning [24,25,29]. Spawning events are usually closely associated with hydrograph peaks [25]. The eggs are very slightly demersal after water hardening [30] and the embryos develop while drifting in the current, kept from settling to the bottom by turbulence. Settling to the bottom is detrimental to the survival of the eggs [30] and sufficient river length and turbulence to keep the eggs from settling is considered a requisite for recruitment [27,31]. The early larvae also drift in the current, though they have the ability to swim vertically and avoid settling if turbulence is insufficient to keep them from settling [32]. Grass carp reproduction has been recently reported from a canal network in the lower floodplain of the Po River where nursery areas are present [33], suggesting bighead carp could become established as well. However, the exact location of spawning remains unclear and no analysis of spawning condition viability has been performed.

Based on their similar spawning requirements, we hypothesized that adult bighead carp could also successfully recruit in the Po River or the associated canal system and that YOY bighead carp would most likely be found in nursery areas where juvenile grass carp had been previously reported. This is of great importance as early detection is paramount to manage potentially invasive species. To supplement the spawning hypothesis, we investigated data on thermal and hydraulic conditions to offer clues to discriminate among potential spawning locations. Finally, if natural reproduction of bighead carp is occurring, an analysis of pathways available within the canal network in relation to viability of egg transport would help ascertain potential locations suitable for recruitment.

## Materials and methods

Field sampling permits have been granted by the Emilia Romagna regional administration and by the water management authority (*Consorzio di Bonifica Pianura di Ferrara*).

### Fish sampling and age analysis

To verify the presence of potential spawners within the canal network, adult bighead carp were sampled during the fall of 2013 and 2014, at stretches of Canal Bianco, Canal Guarda, Canal Acque Alte and Andio, all located upstream of locations where juveniles were collected. Sampling of adult individuals was undertaken at locations where, due to minimum flow in the canals, fish were concentrated in an area of suitable depth for seining (0.6–1.3m) as described in [34]. Seine nets were 2m in height with a 25m mouth and 8mm mesh size, with a 3m long cod-end of 4mm mesh, and were dragged towards a blocking net of 4mm mesh spanning the canal width and depth. After measuring their weight and length, adult individuals were released in nearby canals which had higher water levels. Density was estimated as number of individuals and their biomass per area of the canal, using the area of the canal before water level reduction.

Density of adult individuals was also estimated in the Po River between August and October 2014, using a drifting trammel net 1.80 m high and 150 m long with an inner mesh size of 70 mm and an outer mesh size of 300 mm. In this case, density was estimated as number of individuals and their biomass per area swept by the net.

Investigations for the presence of young-of-the-year (YOY) bighead carp individuals were performed between 2011 and 2015 in similar habitats as used by juvenile grass carp [33]. YOY bighead carp sampling took place in rice field irrigation canals, which are characterized as short sections separated by flow control structures, often with low water velocity but abundant riparian and aquatic vegetation. These canals are situated at a lower elevation than the rice fields, requiring active pumping of the water for irrigation. Sampling was in 1–3 days of September and October of each year and was conducted with a 1m tall and 5m wide seine net (6mm mesh). All juvenile fish were measured (length and weight), transported to the laboratory, identified by morphological discrimination [18] and frozen for further analysis. Silver carp are also present in the area, but extremely rare (˜1% of adult *Hypophthalmichthys* spp., Lanzoni unpublished data) and thus unlikely to be confused with bighead carp.

A total of 29 small bighead carp specimens were captured in October 2012 (n = 7), September 2013 (n = 4) and September 2014 (n = 18). No small specimens were captured in 2011 or in 2015. Unfortunately, 18 specimens were lost due to a refrigerator failure, including specimens from year 2012 (n = 1), 2013 (n = 1) and 2014 (n = 16).

To determine the age and the day-of-birth, lapillus otoliths were extracted, fixed with a resin medium on a glass plate and sanded to expose the nucleus. Daily growth rings were then counted under a stereomicroscope (10x or 20x magnification).

Fertilization dates were estimated for each spawning year, based on date of capture and age of each individual and accounting for egg development time, related to temperature, according to [32]. Given that otolith formation occurs immediately before hatching, hatching dates were estimated to be the age of the fish subtracted from the collection date.

### Reproduction and hydraulic conditions

Onset of Asian carp spawning is thought to be dependent on thermal and hydraulic cues (particularly spikes in the hydrograph; reviewed by [6]), therefore we collected hydraulic data (water temperatures and daily discharges) from both the main river and the canal network. Daily measures of water temperatures were taken by the public water treatment company (*C*.*A*.*D*.*F*. *Ltd)*, in the Po River at the section of Berra municipality (Province of Ferrara) where both the studied siphoning plants of Contuga and Berra are located. Water from the river enters the canal network through these siphoning plants and flows through the system in a low gradient environment, usually with minimal change in temperature (Lanzoni, unpublished data).Temperature data were therefore considered representative of the canal network as well as the main river. Because specific information on bighead carp is lacking, we assumed available information on grass and silver carp would be reasonable surrogates, based on similarities in environments in both native and invaded habitats [27]. The total annual degree-days (hereafter ADD), were calculated to identify whether the thermal thresholds required for maturation (reported to be 2685 ADD during a calendar year; [35]) was reached. Also, the date on which the number of annual degree days above 15°C (hereafter ADD15) reached the minimum for onset of spawning by silver carp (reported to be 655; [36]) was determined for each year. Grass carp has been reported to spawn at temperatures above 20°C in the former Soviet Union [37]. We therefore assumed these three to be valid thresholds for the spawning of bighead carp.

Flooding events are known to trigger bighead carp spawning [6] but the hydrology of the canal network where putative YOY bighead carps were collected is complex. From the Po River, water enters the main irrigation canal, the Canal Bianco, through siphons at the siphoning plants of Contuga and Berra. The siphons are opened and closed according to irrigation needs. Secondarily, the Canal Bianco may receive drainage water from areas upstream of the studied canal network, but only in case of heavy rains, usually in autumn and winter. Siphoning plants let little water through during winter (October-March), thus the water level in the canal network is low throughout these months. Siphons are progressively opened starting from the beginning of April (except in 2013, when they were opened in mid-March), which raises the water level in a flood-like event. Afterwards, the water level in the canal network is more or less constant until the next autumn. Thus, there are both natural (in the main river) and artificial (in the canal network) flood events that could trigger bighead carp spawning. Fish cannot move from the canal network to the Po River, but eggs, larvae and even juveniles drifting in the river could potentially pass through the siphons into the canal network. For a detailed description of migration pathways available to bighead carp see [33].

Po River water level was manually measured daily in Pontelagoscuro, roughly 40 km upstream of the siphoning plants, and was converted to discharge through a model developed by the water management authority (*Consorzio di Bonifica Pianura di Ferrara*, unpublished). Since no tributaries and water diversions are present between Pontelagoscuro and the plant of Contuga, this discharge was representative of the stretch of the main river where bighead carp spawning could potentially occur upstream of the siphons.

Data on discharge in the first section of the canal network were obtained from the water management authority (*Consorzio di Bonifica Pianura di Ferrara*) at the Contuga and Berra plants, the two main irrigation plants feeding water to Canal Bianco and the section where YOY were found. These data were measured daily at the siphons. Water velocity (m/s) was derived from discharge for each of the main canal derivations, based on canal sections, and data were verified with experimental measurements performed at main and secondary sections, using a current meter (Open Stream Current Velocity Meter, 2100, Swoffer Instruments Inc, Seattle, WA, USA). Water velocity was measured directly during peak flow in 2016 for each section of the putative egg pathways. The meter was mounted on a measuring pole with centimetric resolution, equipped with a modified propeller for low flow conditions.

**Fig 1:**
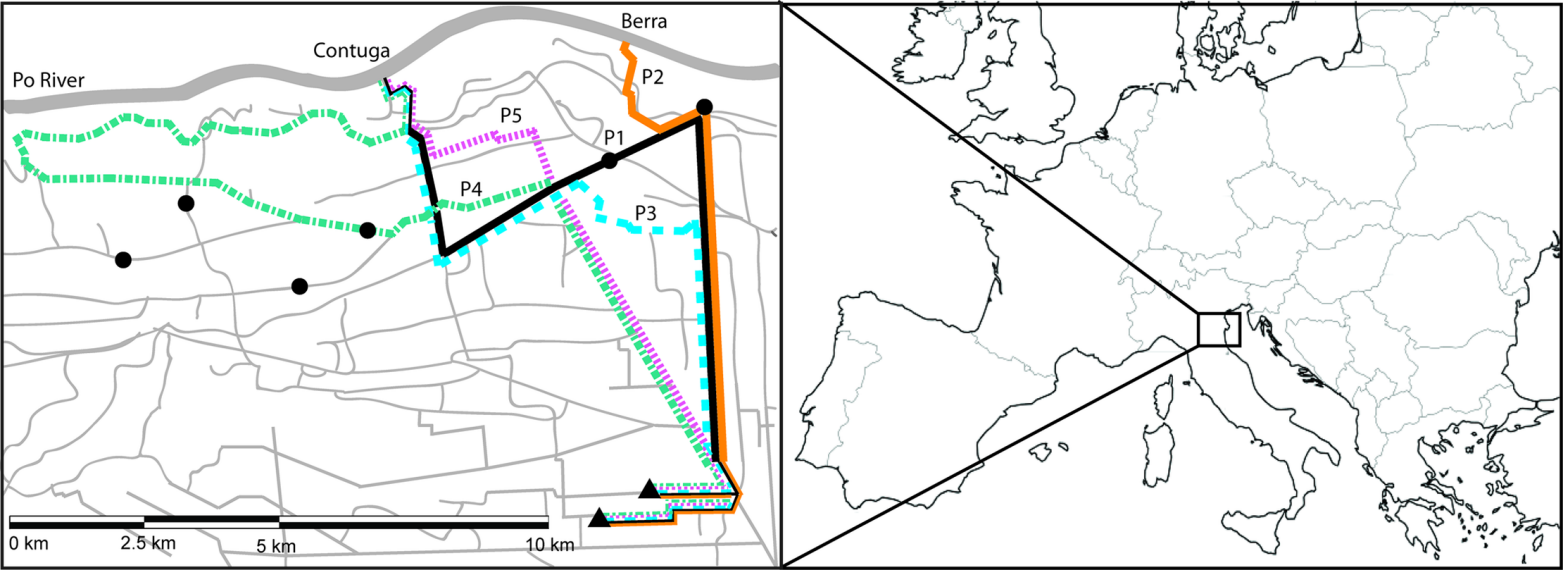
Schematic representations of five potential egg pathways within the canal network. Po River, at the top of the map, and canals of the network are presented with solid grey lines. Solid and dashed color lines represent available pathways from the Contuga (P1, in solid black, and P3–P5, in blue long dashes, green dash and dots and pink short dashes, respectively) and Berra (P2, in solid orange line) siphoning stations to the locations where YOY individuals were captured, represented with black triangles. Sampling locations of adult bighead carp are represented with black circles.

Because adult fish in the canal network cannot migrate upstream of the siphons, we assumed that if spawning occurred within the canal system, it would occur in the turbulent area immediately downstream of the siphon site. We estimated all 5 potential egg pathways leading from the putative spawning location to the locations where YOY were captured (Fig 1). For each of these pathways, based on our hydrographic data, we calculated length and water transit times for every year when YOY were captured. To assess the viability of all pathways we then compared egg hatching times to water transit times. We also checked whether water velocity would be sufficient to keep eggs from sinking to the bottom, which can potentially prevent eggs from hatching.

## Results

A total of 350 adult bighead carp were sampled in the canal network between 2013 and 2014 (average weight 13.2 kg, maximum length 102.5 cm and maximum weight 21 kg). Average adult bighead carp density was 2.7 fish/ha and 45.2 kg/ha, but these values were also extremely variable among years and locations (1.0–7.0 fish/ha; 11.6–127.6 kg/ha). Density of bighead carp in the Po River was much lower than in the canal network (0.1 fish/ha; 1.8 kg/ha).

**Fig 2:**
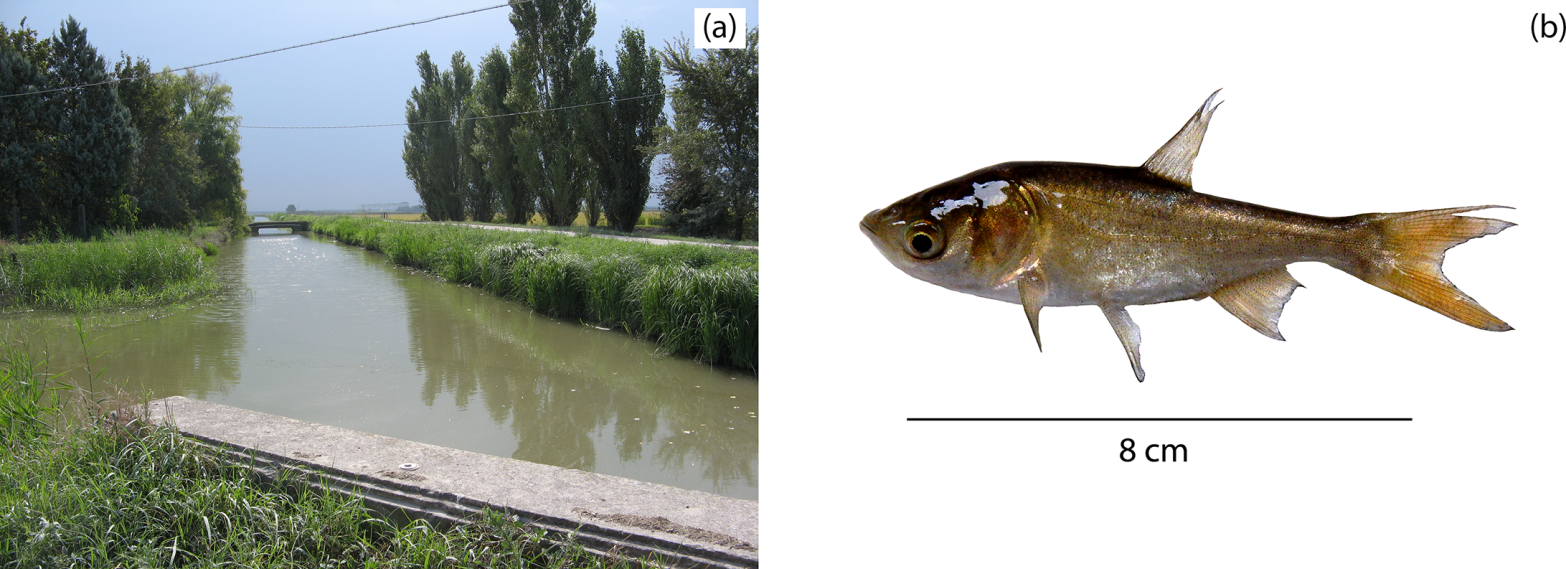
(a) the sampling location where all juvenile individuals were found and (b) one of the YOY bighead carp sampled during this study.

A total of 29 YOY bighead carp were captured between 2012 and 2014 at two locations in the irrigation canals (Fig 2). No YOY bighead carp were captured in 2011 or 2015. Young bighead carp were 7.4–13.1 cm long (TL) and weighed 9.5–12.7 g. The bighead carp in the subsample analyzed for age and DOB (n = 11) were 7.4–12.9 cm (TL) and weighed 9.5–11.1 g.

Age estimates from otoliths confirmed that all analyzed specimens were YOY. Daily growth ring analysis estimated that specimens from all years were 94–100 days old, placing their fertilization and hatch dates in mid-to-end-June, depending on the year (Table 1).

**Table 1:** Catch date, total length (TL), weight (g), age (in days) as well as hatch and fertilization dates for all specimens of YOY bighead carp examined in this study.

Catch date	Total length (cm)	Weight (g)	Age (days)	Hatch date	Fertilization date
02.10.2012	7.9	10.2	97	27.06.2012	26.06.2012
02.10.2012	7.9	9.5	96	28.06.2012	27.06.2012
02.10.2012	8.7	10.1	98	26.06.2012	25.06.2012
02.10.2012	11.5	11.1	100	24.06.2012	23.06.2012
02.10.2012	11.7	10.9	97	27.06.2012	26.06.2012
02.10.2012	11.4	10.8	100	24.06.2012	23.06.2012
16.09.2013	8.8	10.8	94	14.06.2013	13.06.2013
16.09.2013	12.1	10.4	97	11.06.2013	10.06.2013
16.09.2013	12.9	10.6	98	10.06.2013	9.06.2013
20.09.2014	7.4	9.6	94	18.06.2014	17.06.2014
20.09.2014	11.8	10.5	100	12.06.2014	11.06.2014

None of the fertilization dates corresponded with the mid-March to early-April flooding of the canal network, so we excluded those water level variations as cues for bighead carp spawning within the canal network.

The ADD threshold for maturation was always reached, both in non-spawning and spawning years, by first half of July. Overall, ADD were about 5000, meeting and exceeding annual thermal requirements for maturation.

**Fig 3:**
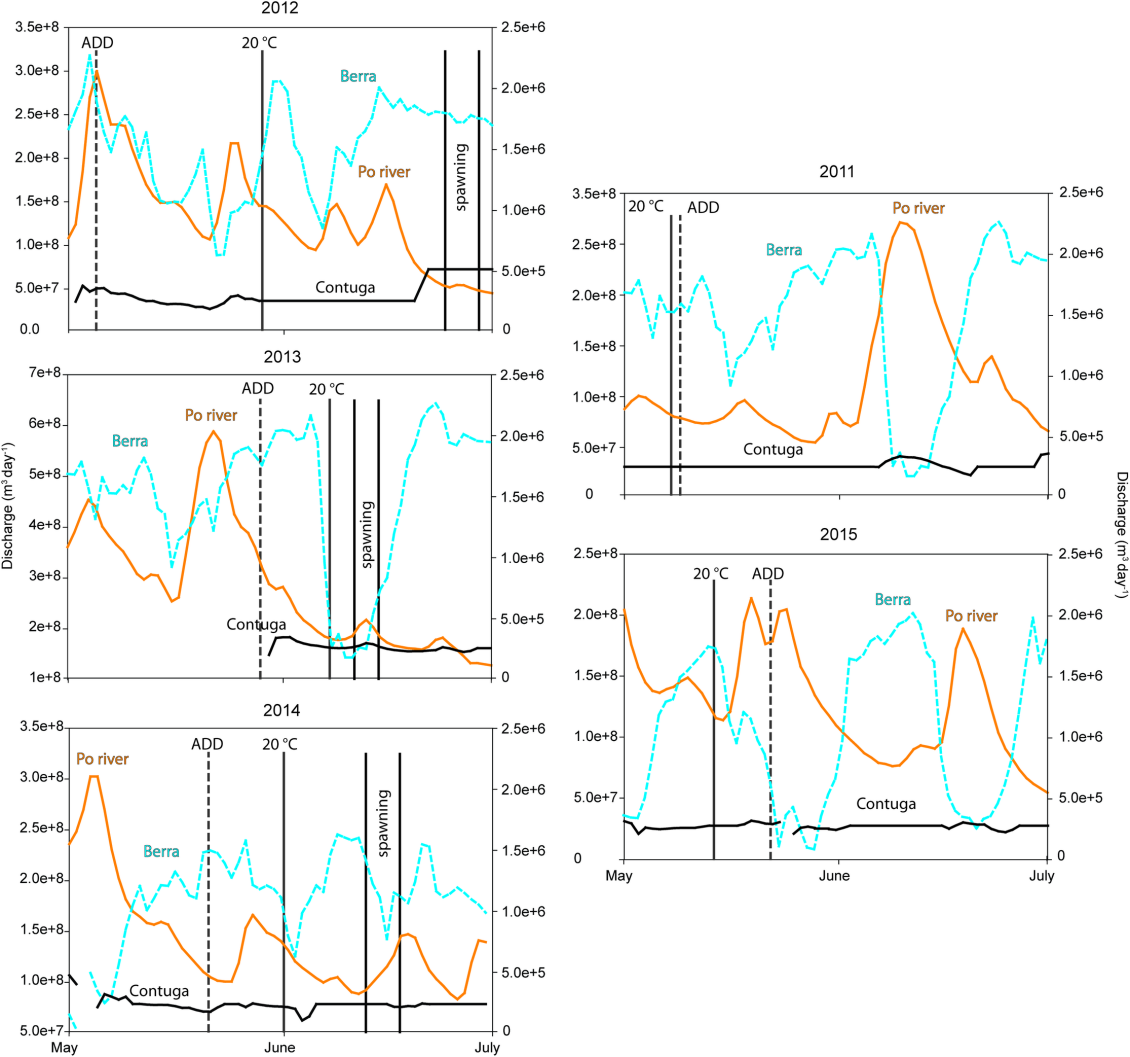
Hydrographs of the discharge in the main river (in solid orange, left Y axis), the Contuga siphon (in solid black, right Y axis) and the Berra siphon (in dashed blue, right Y axis). Vertical lines represent the ADD15 (dashed grey) threshold, first day of 20°C (solid gray) and estimated fertilization dates (solid black).

The ADD15 and 20°C thresholds for onset spawning were always met, in that order, prior to estimated fertilization dates, in years when bighead carp YOY were captured (2012–2014) whereas in other years (2011 and 2015), the 20°C threshold was reached before the ADD15 threshold (Fig 3).

In 2012, bighead carp spawning occurred in late June, during a moment of maximum flow from the Contuga plant but minimum and decreasing flow in the Po River and Berra plant. In 2013, bighead carp spawning also occurred during a peak in flow from the Contuga plant but concurring with a small peak in the main river flow. Similarly, in 2014 spawning occurred in mid-June, during a rising hydrograph in the main river.

Estimated egg hatching times were 20, 34.3 and 24.3 hours for 2012, 2013 and 2014, respectively. These hatching times were nearly all compatible with transit times within the different available egg pathways within the canal network and a viable pathway was always available in each year (Table 2).

**Table 2:** Transit time difference (water transit time, minus egg hatching time) for each available egg pathway within the canal network, for every year when YOY were captured. Bold values highlight incompatible times of transit (negative values indicate that eggs would hatch beyond the end of the pathway).

		2012		2013	2014	
	Total length (km)	Transit time difference (hours)				
Pathway 1	18.75	20.64		6.38	16.71	
Pathway 2	11.41	3.97		**-9.65**	0.03	
Pathway 3	17.02	13.35		**-0.91**	9.43	
Pathway 4	31.63	201.13		186.84	196.95	
Pathway 5	13.97	16.78		2.50	12.68	

All pathways included current velocity bottlenecks, with water velocity dropping as low as 0.03 m s^-1^. A common bottleneck was at the convergence of all Pathways, in Canale Leone, but this was limited to a short, terminal stretch for pathways 1–3. Conversely, Pathways 4 and 5 shared a greater length of the slow-flowing Canale Leone and Pathway 4 had low speed bottlenecks already in its initial stretch. Overall, Pathway 2 provided the closest matches with hatching times in all years.

## Discussion

Large adult bighead carp and, in some years, YOY are present in the canal network, indicating successful recruitment. To our knowledge, this is the first account of bighead carp natural reproduction in West Europe. All thermal thresholds were met and seemed to corroborate the viability of spawning conditions and fertilization dates estimated from otoliths. Natural reproduction of grass carp, which has similar reproductive requirements, has been previously confirmed within the study area [33].

No inspection was conducted on the gonad development of the adult fish sampled in this study but bighead carp typically mature at lengths of 55–70 cm and weight around 3 kg [38] therefore the population in the canal network is composed in large part by mature fish that could be potential spawners. Bighead carp capture rates in the canal network were variable, likely reflecting a non-uniform spatial distribution of individuals, and marked differences in individual size. However, the highest density sampled in the canals (242.4 kg/ha, for 11.5 fish/ha) indicates that large concentrations of spawning-size fish are possible and more likely to occur here than in the Po River, where densities were markedly lower. Higher densities could be reached within the canal network during the spawning season, if fish are grouped at impassable migration barriers such as the siphoning plants.

The question of whether the collected YOY bighead carp were spawned in the Po River or within the canal network itself is intriguing but cannot be fully resolved. In 2013, hydrograph peaks in both the Po River and in the canal coincided with the estimated egg fertilization dates, which is consistent with the preference of Asian carps for spawning during hydrograph peaks, but provides no clues as to whether spawning occurred in the Po River or in the canal. While in 2014 the rising hydrograph in the river could be the cue for spawning, the time of spawning in 2012, following an increased hydrograph from the Contuga plant, but not in the river, lends credit to a hypothesis that bighead carp spawned within the canal network. The system has very unusual conditions for spawning and recruitment by an Asian carp and if spawning did occur within the canal system it would constitute the only record of Asian carp spawning within a canal system other than the extremely large and river-like Karakum Canal in Turkmenistan (1375 km long and navigable for most of its length; [39]). However, it cannot be confirmed with certainty that the spawning event occurred within the canal network, because some Asian carp spawning (though reduced in intensity) does sometimes occur outside periods of hydrograph peaks [40,41].

Because of the intriguing potential that spawning may have occurred within the canal system itself, we investigated five pathways within the complex canal system and their potential for egg transport that would support survival and recruitment (Fig 1). While some pathways (especially 2 and 3) had transport times close to hatching times in all years, some low velocity areas existed in the pathways where eggs may not have been adequately carried by the current. Pathway 4, in particular, was not likely to be viable, while Pathway 2 transit times were likely adequate in 2013 and 2014. Furthermore, hatching times in 2013 indicated that eggs in Pathway 2 and 3 could have hatched before they reached the potential low velocity bottleneck of Canale Leone, in the terminal stretch of the pathways. This estimation of drift distance is rather coarse but might work in these canal systems, with relatively simple hydraulics. However, a better understanding and modeling of these hydraulics would be needed to accurately estimate where spawning occurs.

It has been reported that a minimum water velocity of 0.15 m s^-1^ is necessary to keep Asian carp eggs adrift even if higher turbulence resulting from shallow depth plays a relevant role in keeping eggs from settling [42]. The consequence of failing to meet this requirement is settlement to the sediment, which ultimately increases the chances of death for the embryo, especially if the egg is fully or partially buried [43]. In our study system, velocities below this threshold were detected, at least in Canale Leone, where all pathways ultimately converge. However, Asian carp eggs are large and mechanically resistant, and it may be that if the partially-hardened egg does not experience a complete stop, it could continue downstream, occasionally contacting the bottom without stopping its drift. This mechanism is possible in our study system where low sedimentation rates are accompanied by simplified canal morphology. Some of these canals have smooth hard bottoms and banks, either concrete or hard clay, free of macrophytes and large plant debris which decreases turbulence. These characteristics also decrease the chance of eggs becoming lodged and ultimately buried. Furthermore, [43] found that mortality rates of settled eggs were much reduced if the eggs settled when they were near hatching, and that some eggs survived in some conditions even if they settled early in development. If eggs fertilized at the siphons reach the low velocity zone in Canale Leone, where all Pathways converge, they would have been very near the hatching (or even hatched, as in 2013) and thus they could potentially survive this bottleneck, unless they were buried by sediment in that short period. Oxygenation of the water column might still constitute a bottleneck, because some of the canals in the study area occasionally experience low-oxygen events during summer [34]. However, low oxygen concentrations typically occur later in the summer (July, August) and thus may not be relevant to the peak reproductive season of bighead carp. Thus, reproduction of bighead carp within the canal system cannot be ruled out.

All thermal thresholds for maturation (ADD) and onset of spawning (ADD15 and 20°C) were met in each year. Notably, in 2011 and 2015, when no evidence of reproduction was found, the 20°C threshold was reached first, contrary to years when reproduction was confirmed, when ADD15 was reached first. However, we do not know if this order is relevant to successful recruitment.

There was no substantial difference between hydrographical and thermal regimes of years when recruitment was detected and years in which it was not detected. It is possible that reproduction could have been successful in all years and that YOY were simply not detected, but YOY of grass carp were detected with similar sampling methods and locations [33] which suggests that sampling was at least appropriate. Bighead carp and grass carp have similar spawning requirements (e.g. [25]) so it is likely that if one species is able to reproduce, the other will also be able [44]. However, natural reproduction of grass carp in the area was reported to be fairly consistent from 2010–2015 [33], so there might be species-specific mechanisms yet unknown. It has been reported that, in China, grass carp are somewhat more plastic than the other Asian carps in terms of spawning requirements [45], which might explain the difference in recruitment consistency, but no literature is yet available to adequately evaluate species-specific differences [30,32,43]. Therefore, further studies would be needed to unravel the ultimate causes of differential reproduction success.

After flooding of the canals in spring time, because of artificial regulation of flow, there are low fluctuations of water level in the canal network until winter, unlike in natural rivers where increased flow is linked to rising water levels. While in the literature it is widely reported that one of the primary spawning cues for the species is a rising hydrograph [25], fish might only perceive variations in flow intensity and thus might not be able to gauge the height of the water level at any given time. Some spawning (usually with reduced intensity) has already been shown to occur during periods of stable or low flow in the introduced range in North America [40,41]. Thus, bighead carp could respond to artificial variations in flow and reproduce in systems previously considered unsuitable. Furthermore, bighead carp and grass carp spawning is generally thought to occur in rivers larger than these canals, but Coulter et al. [40] described substantial plasticity in bighead and silver carp spawning in its introduced range in the USA, including much smaller rivers than previously reported, and a spawning season protracted into early fall. Our results clearly support previous findings that the limitations to successful reproduction most commonly reported in the literature for this species are not rigid.

### Summary

Bighead carp were found to have recruited in northeastern Italy, the first time that bighead carp are known to successfully recruit in west Europe. The increase of flow in the canal network, but not in the river, during the time of bighead carp spawning in 2012, lends credence to a hypothesis that bighead carp spawned in the canal rather than in the river. Egg drift pathways within the canal system were congruent with recruitment within the canal system. Further studies are needed to determine whether bighead carp spawned in this unusual location. Nevertheless, the presence of YOY in West Europe canals shows bighead carp are able to successfully recruit in an area previously considered inadequate for the species. The potential invaded range in West Europe should be reconsidered taking into account the possibility of successful reproduction in sub-optimal conditions.

It would be important, in order to formulate information-based management plans, to quantitatively assess the current and projected effects of bighead carp and other Asian carps on ecosystems of Western Europe. Because both adults and YOY have been captured repeatedly, the species has clearly established in the basin, although the precise location of spawning has not been identified. Bighead carp have repeatedly proven to have the potential to become invasive in other areas, thus their establishment in Italy is of concern.

## Supporting information

S1 Table Date, location and dimensions of YOY bighead carp individuals captured in the canal network (yellow and orange highlights mark individual samples that were lost due to freezer failure). (XLSX)

S2 Table Estimated egg hatching times and transit times in the different pathways for egg drift analyzed in this study. (XLSX)

S3 Table Thermal and hydraulic thresholds, egg release dates and daily discharges of the Contuga and Berra siphons as well as of the Po river (data underlying Fig 3). (XLSX)
